# Multi-Task Classification and Segmentation for Explicable Capsule Endoscopy Diagnostics

**DOI:** 10.3389/fmolb.2021.614277

**Published:** 2021-08-19

**Authors:** Zishang Kong, Min He, Qianjiang Luo, Xiansong Huang, Pengxu Wei, Yalu Cheng, Luyang Chen, Yongsheng Liang, Yanchang Lu, Xi Li, Jie Chen

**Affiliations:** ^1^ School of Electonic and Computer Engineering, Peking University, Shenzhen, China; ^2^ Department of Gastroenterology, Peking University Shenzhen Hospital, Shenzhen, China; ^3^ Peng Cheng Laboratory, Shenzhen, China; ^4^ Sun Yat-sen University, Guangzhou, China; ^5^ Pennsylvania State University, Philadelphia, PA, United States; ^6^ Harbin Institute of Technology (Shenzhen), Shenzhen, China; ^7^ Beijing Normal University-Hong Kong Baptist University United International College, Zhuhai, China

**Keywords:** Capsule endoscopy, Multi-task learning, Explicable, Crohn’s disease, Auxiliary diagnosis

## Abstract

Capsule endoscopy is a leading diagnostic tool for small bowel lesions which faces certain challenges such as time-consuming interpretation and harsh optical environment inside the small intestine. Specialists unavoidably waste lots of time on searching for a high clearness degree image for accurate diagnostics. However, current clearness degree classification methods are based on either traditional attributes or an unexplainable deep neural network. In this paper, we propose a multi-task framework, called the multi-task classification and segmentation network (MTCSN), to achieve joint learning of clearness degree (CD) and tissue semantic segmentation (TSS) for the first time. In the MTCSN, the CD helps to generate better refined TSS, while TSS provides an explicable semantic map to better classify the CD. In addition, we present a new benchmark, named the Capsule-Endoscopy Crohn’s Disease dataset, which introduces the challenges faced in the real world including motion blur, excreta occlusion, reflection, and various complex alimentary scenes that are widely acknowledged in endoscopy examination. Extensive experiments and ablation studies report the significant performance gains of the MTCSN over state-of-the-art methods.

## 1 Introduction

Deep learning and convolutional neural networks have recently shown outstanding performances for visual recognition and semantic understanding [Krizhevsky et al. (2012); Simonyan and Zisserman (2014); He et al. (2016); Huang et al. (2017); Long et al. (2015)]. The representation learning capacity of convolutional neural networks has also been successfully applied to medical image analysis and recognition in gastrointestinal endoscopy [Ronneberger et al. (2015); Le et al. (2019); Hwang et al. (2020)]. Crohn’s disease [Podolsky (1991); Baumgart and Sandborn (2012)] is an inflammatory bowel disease (IBD), and its signs and symptoms range from mild to severe. It usually develops gradually but sometimes will come on suddenly, without warning. While there is not a known cure for Crohn’s disease, early detection and preventative therapies will greatly reduce its signs and symptoms and even bring about long-term remission. Because the small intestine and colon can be affected by Crohn’s disease, capsule endoscopy is the gold standard to examine the midsection of the gastrointestinal tract.

A major challenge in capsule gastroscopy is that the procedure will output a video of several hours which suffers from complicated gastrointestinal environmental challenges, such as excreta occlusion, motion blur, and light scattering, wasting plenty of time for professional gastroenterologists to find out the location of lesions [Min et al. (2019)]. Although several software enhancements, including Quick-View (Medtronic, Minneapolis, MN, United States) and Express View (CapsoVision, Inc., Saratoga, CA, United States), attempt to overcome these drawbacks, their performance is insufficient for use in clinical practice because of their limited accuracy and unexplicable output [Hwang et al. (2020)]. To assist gastroenterologists to locate Crohn’s lesions explicably and precisely, we introduce a dataset named the Capsule-Endoscopy Crohn’s Disease dataset, a large-scale Crohn’s gastrointestinal image dataset for clearness degree (CD) and tissue semantic segmentation (TSS) which will greatly help doctors understand the classification results. The proposed dataset covers 467 images in real-world scenarios.

In the meanwhile, we propose a multi-task learning (MTL) scheme, which combines pixel-level segmentation and global image-level category classification. The proposed architecture is based on a fully convolutional image-to-image translation scheme, which enables efficient feature sharing between image regions, and fast prediction. A novel cross fusion module is proposed to mitigate the gap between different foci of classification and segmentation tasks. We evaluate our model on the proposed dataset, with clearness degree classification and tissue segmentation with eight classes. We show that through joint training, the model is able to learn shared representations that are beneficial for both tasks. Our method can be seen as a generalization of approaches relying on detection annotations to pre-train the deep model for classification purposes. We show that our joint training of classification and segmentation enables a better cooperation between tasks.

## 2 Related Work

### 2.1 Image Classification

Since AlexNet [Krizhevsky et al. (2012)], deep convolutional neural networks have dominated image classification. With this trend, research has shifted from engineering handcrafted features to engineering network architectures. VGG-Net [Simonyan and Zisserman (2014)] proposes a modular network design strategy, stacking the same type of network blocks repeatedly, which simplifies the workflow of network design and transfer learning for downstream applications. Built on the success of this pioneering work, He et al. (2016) introduced an identity skip connection which alleviates the difficulty of vanishing gradient in the deep neural network and allows for network learning deeper feature representations. Reformulations of the connections between network layers [Huang et al. (2017)] have been shown by DenseNet to further improve the learning and representational properties of deep networks. DenseNet has become one of the most successful CNN architectures which has been adopted in various computer vision applications.

### 2.2 Semantic Segmentation

With the great success of deep learning in high-level vision tasks, numerous semantic segmentation approaches [Long et al. (2015); Ronneberger et al. (2015); Zhao et al. (2017); Chen et al. (2018)] are beneficial for CNNs. Long et al. (2015) first introduced fully convolutional networks (FCNs) for semantic segmentation which conduct pixel-wise classification in an end-to-end fashion. While U-Net was introduced by Ronneberger et al. (2015), which concatenates the up-sampled feature maps with feature maps skipped from the encoder.

Due to the precise pixel-level representation, deep learning–based semantic segmentation has been widely adopted in lesion and tumor segmentation, helping doctors get an accurate and explicable diagnosis. Li et al. (2018) proposed H-DenseUNet for liver and liver tumor segmentation. A modification to U-Net was proposed by Zhou et al. (2019), named UNet++, which is applied to a variety of medical datasets for segmentation tasks.

### 2.3 Multi-Task Learning

**FIGURE 1:**
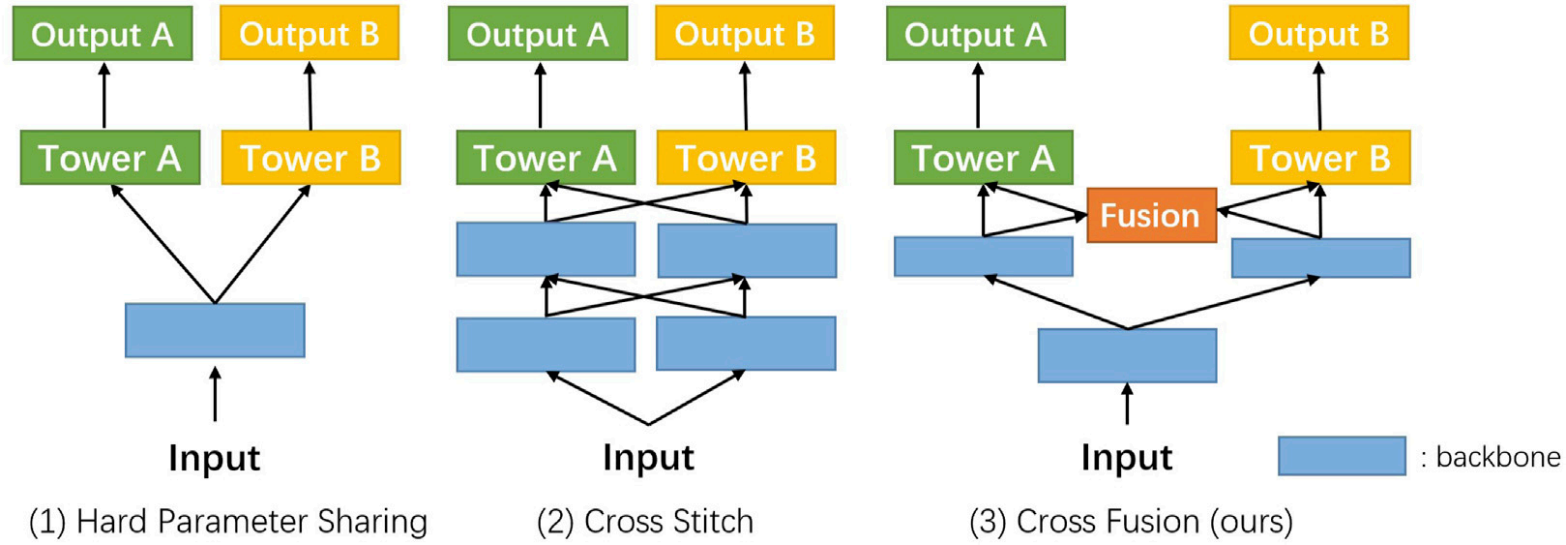
Evolution routing map of the multi-task network structure. Blue rectangles represent the shared layers, like the backbone. Green and yellow ones denote the different task branches. The orange block is the cross fusion module introduced by us in this work.

Multi-task learning [MTL, Caruana (1997)] is often applied when related tasks can be performed simultaneously. Many MTL methods [Jalali et al. (2010); Misra et al. (2016); Gebru et al. (2017); Strezoski et al. (2019)] have achieved great success in a variety of computer vision tasks. In the medical domain, some recent works also focus on combining classification and segmentation into a joint framework. Yang et al. (2017) proposed a multi-task DCNN model for skin lesion analysis. Multi-task classification and segmentation was proposed by Le et al. (2019) for diagnostic mammography. In the recent COVID-19 pandemic, multi-task learning was applied in CT imaging analysis by Amyar et al. (2020). MTL schemes are based on the assumption that the difficulty of classification and segmentation tasks is the same. But in the real scenes, especially in the small intestine, classification is much simpler than segmentation tasks. Some pioneers have proposed a weighted loss design [Kendall et al. (2018)] and attention module [Liu et al. (2019)] to balance different types of tasks. As shown in Figure 1, the evolution of MTL tends to bring more precise control on fusion between different tasks. We dive into this problem and introduce our solution to it.

## 3 Proposed Method

To assist the gastroenterologists in capsule endoscopy examination, both precision and interpretability are necessary. Following the previous methods [Le et al. (2019)], we model the precision and interpretability tasks into classification and segmentation tasks. Our proposed multi-task framework shows that joint training of classification and segmentation enables a better cooperation between tasks.

**FIGURE 2:**
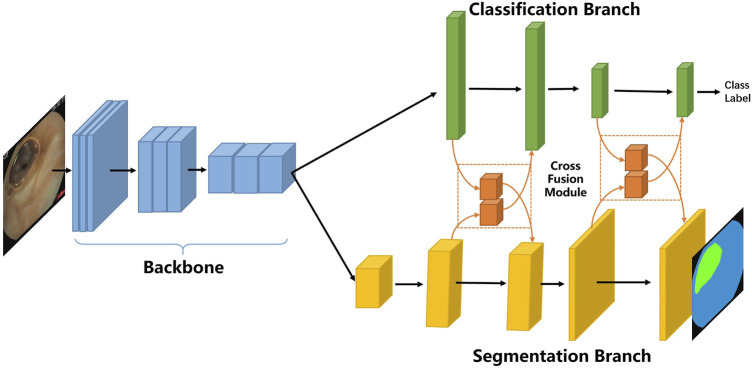
Multi-task classification and segmentation network (MTCSN) architecture.

In the following, we first describe the overall framework of our proposed multi-task classification and segmentation network (MTCSN), shown in Figure 2. Specifically, a backbone is adopted to extract the representations of the input image which are further used to generate the class label and segmentation map. Next, we introduce the cross fusion module, the key elements of the MTCSN, to alleviate the misalignment between classification and segmentation. Finally, we dive into the inherent problem in the multi-task learning training strategy and introduce our object function.

### 3.1 Network Architecture

As shown in Figure 2, the proposed multi-task classification and segmentation network first utilizes a backbone to extract local features. The backbone we adopted includes different depths of ResNet or DenseNet. Following feature extraction, we design two multi-task branches which are the classification branch for image clearness degree measuring usability and the segmentation branch for tissue segmentation producing explicable visualization to help doctors understand the whole image. The classification branch is mainly constructed by fully connected layers, and the segmentation branch is based on an image-to-image scheme enabling efficient feature computation in each region but also sharing computation from all regions in the whole image in a single forward pass. In addition, we can still process input images with high spatial resolution.

### 3.2 Cross Fusion Module

Our network mainly focuses on two tasks, classification and segmentation. In the prevailing pattern of MTL, two branches have been trained separately for these tasks following the shared backbone for feature extraction [Figure 1]. Because the classification task and segmentation task place different emphasis on feature extraction, performance degeneration is foreseeable and needs to be resolved.

**FIGURE 3:**
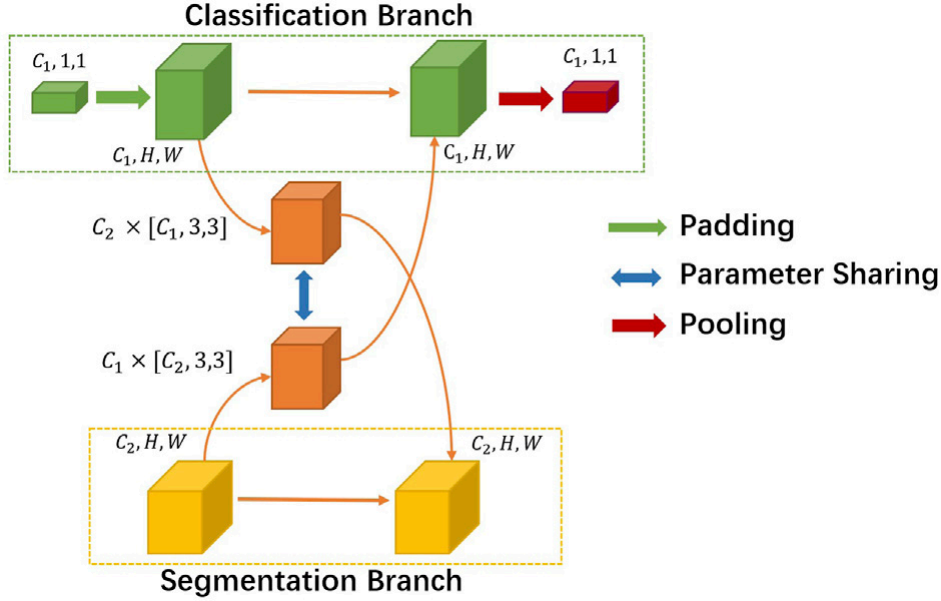
Details of the cross fusion module.

Instead of designing two parallel backbones [Misra et al. (2016)], we set our sights on efficiently exploiting the interaction between the two tasks’ branches. We introduce a novel non-linearity cross fusion module which learns the extent of sharing, as illustrated in Figure 3.

After global average pooling, the classification branch feature’s usual shape is [*C*_1_, 1, 1], where *C*_1_ denotes the number of channels. While the segmentation branch feature’s shape is [*C*_2_, *H*, *W*], *C*_2_ is usually not the same as *C*_1_. First, we mold the classification feature into the same shape of segmentation. Then, we utilize a sharing parameter non-linearity transformation matrix *M* to learn the joint representations and extent of fusion automatically. In our experiment setting, *M* is formulated as a parameter matrix of the convolution layer. More precisely, the process of the cross fusion module can be formulated as

$$\left\{\begin{array}{c} {\tilde{X}}_{cls}=X_{cls}+Pool\left(M\left(X_{seg}\right)\right),\;\\ {\tilde{X}}_{seg}=X_{seg}+M^{T}\left(Pad\left(X_{cls}\right)\right),\; \end{array}\right.$$ (1)

where *X*_*cls*_ and *X*_*seg*_ denote the classification and segmentation feature inputs to cross fusion. *M* denotes the non-linearity transformation matrix, and *M* ^*T*^’s dimension order is different. The output of cross fusion is ${\tilde{X}}_{cls}$ and ${\tilde{X}}_{seg}$. The network can automatically decide to make certain layers task-specific by setting a lower weight to the matrix or choosing a more shared representation by assigning a higher value to it.

### 3.3 Object Functions

In general multi-task learning with *K* tasks, input *X*, and task-specific labels *Y*_*i*_, *i* = 1, 2, … , *K*, the loss function is defined as

$$L_{all}=\overset{K}{\underset{i=1}{\sum }}\lambda_{i}L_{i}\left(X,Y_{i}\right).$$ (2)

With task weightings *λ*_*i*_, $L_{all}$ is the linear combination of task-specific losses $L_{all}$. We study the effect of different weighting methods on our multi-task learning approaches. The overall object function of the MTCSN is composed of two parts:

• For the classification task, we apply a class-wise cross-entropy loss for each predicted class label from a softmax classifier:

$$L_{cls}=\Phi_{CE}\left(X_{cls}^{\prime },X_{cls}\right)+\alpha L_{consistency},$$ (3)

where

$$L_{consistency}=\sum \Phi_{MSE}\left(X_{i}^{\prime },X_{i}\right).$$ (4)

Here, $X_{cls}^{\prime }$ is the predicted classification category. $X_{i}^{\prime }$ and *X*_*i*_ are the features before and after cross fusion in the classification branch. *Φ*_*CE*_ and *Φ*_*MSE*_ are the cross-entropy loss and MSE loss functions, respectively. We empirically set the weight *α* = 0.1 in network training.

• For the segmentation task, we apply a pixel-wise cross-entropy loss for each predicted class label from a softmax classifier:

$$L_{seg}=\Phi_{CE}\left(X_{seg}^{\prime },X_{seg}\right),$$ (5)

where $X_{seg}^{\prime }$ represents the predicted segmentation maps.

## 4 Experiments and Discussion

### 4.1 Datasets and Tasks

Though Crohn’s disease diagnosis is reliable using capsule endoscopy, there is no such open-sourced image dataset for further study so far. So, we build the first Capsule-Endoscopy Crohn’s Disease dataset which includes 15 patients and 164 video clips. The dataset will improve the efficiency and accuracy of gastrointestinal endoscopy and help gain a better understanding of this disease.

**FIGURE 4:**
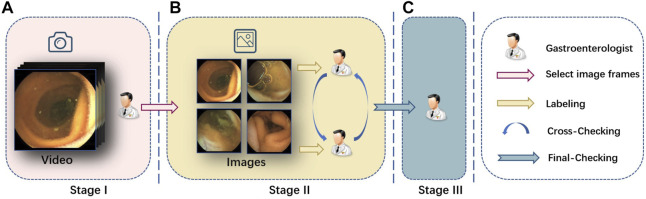
Labeling pipeline we adopted. **(A)** In stage I, we invite several senior gastroenterologists to pick up the video clips of interest, and then we transform them into frames. **(B)** In stage II, we further invite another two gastroenterologists to label the clearness degree of the frames and semantic masks for each part. Cross validation is performed at the same time. **(C)** In stage III, a senior expert checks the labeling and makes the final decision on the annotations.

We divide the annotation process into three stages, and the gastroenterologists are divided into three teams corresponding to these three stages, as shown in Figure 4.

In the first stage, gastroenterologists collect the source capsule endoscopy videos from the database center of the hospital, and all the 15 patients’ capsule endoscopy videos are filmed by MOMO Wireless Capsule Endoscopy JS-ME-I. Then, we invite several gastroenterologists to pick up the video clip of interest from the full examined videos whose length normally lasts 3–4 h. Finally, we take screenshots from these video clips by a fixed frame rate and get images for follow-up stages.

In the second stage, two gastroenterologists are introduced to label the previous screenshots, respectively, at the pixel level and image level. They first classify the image into three clearness degrees according to adequacy assessment [Brotz et al. (2009)] and then segment the scenes into given categories. In the meantime, one gastroenterologist’s annotations will be annotated by another doctor without knowing it, and divergence will be handed over to the third stage’s chief to decide.

In the third stage, all revised images are submitted to the chief and expert gastroenterologist in stage III for final-checking. All the data are anonymized for privacy protection.

Here are the statics of the two tasks in our dataset:

1) Task 1: Clearness degree classification
2) Task 2: Tissue segmentation for precise understanding of the image

The total number of annotation images is 467, and we split the dataset into training, validation, and testing datasets strictly by stratifying the sampling in the clearness categories. There are 372 images in the training dataset, 47 images in the validation dataset, and 47 images in the testing dataset. The statistic of basic attribute of our proposed datasets have been shown in Tables 1, 2.

**TABLE 1:** Details about the classification category distribution.

Category	Number
Clearness	323
Blur	101
Invisible	42

**TABLE 2:** Statistics of segmentation annotation in the dataset.

Category	Number	Category	Number
Clear tissue	361	Invisible by bubble	196
Blur tissue	128	Invisible by excreta	212
Lesion	91	Clear bubble	46
Hole	153		

### 4.2 Evaluation Metrics

The classification results are evaluated by accuracy, precision, recall, and F1 score. A classic classification problem has four possible outcomes, true positive (TP), false positive (FP), false negative (FN), and true negative (TN). Accuracy is the fraction of predictions our model got right. Precision measures the proportion of actually correct positive identifications, and recall answers the proportion of actual positives identified correctly. F1 is an overall measure of a model’s accuracy that combines precision and recall:

$$\begin{array}{c} \begin{array}{cc} \text{ Accuracy } & =\frac{TP+TN}{TP+TN+FP+FN},\\ \text{ Precision } & =\frac{TP}{TP+FP},\\ \text{ Recall } & =\frac{TP}{TP+FN},\\ F_{1} & =2\times \frac{{\text{ Precision }}^{*}\text{ Recall }}{\text{ Precision }+\text{ Recall }}. \end{array} \end{array}$$ (6)

The segmentation results are evaluated using the Jaccard index, also known as Intersection-over-Union (IoU). The IoU is a measure of overlap between the area of the automatically segmented region and that of the manually segmented region. The value of IoU ranges from 0 to 1, with a higher value implying a better match between the two regions. Pixel-wise accuracy is also used for evaluation.

### 4.3 Experimental Results

In this section, we first evaluate several baselines in our Capsule-Endoscopy Crohn’s Disease dataset, respectively, on classification and segmentation tasks. Then, we evaluate our proposed method on two types of tasks. The implementation of our method was done using PyTorch. The model was performed on an Nvidia RTX 2080Ti GPU with 11 gb. The batch size is set to 8, and all images are resized to 240 ∗ 240 to speed up training.

#### 4.3.1 Baselines Results

• **Single Task, Classification Task**. We evaluate two different types of models on our classification problem. Table 3 shows that existing CNN-based classification models already have an acceptable accuracy, precision, and recall score. On account of the scale of datasets and shape of the input image, a simpler and shallower classification model is preferred.

**TABLE 3:** Three-class clearness degree baseline classification results in the CECD dataset.

Classification method	Accuracy	Precision	Recall
ResNet-50	84.0	72.57	72.81
ResNet-101	81.9	69.67	71.41
DenseNet-121	86.7	73.48	73.72

**FIGURE 5:**
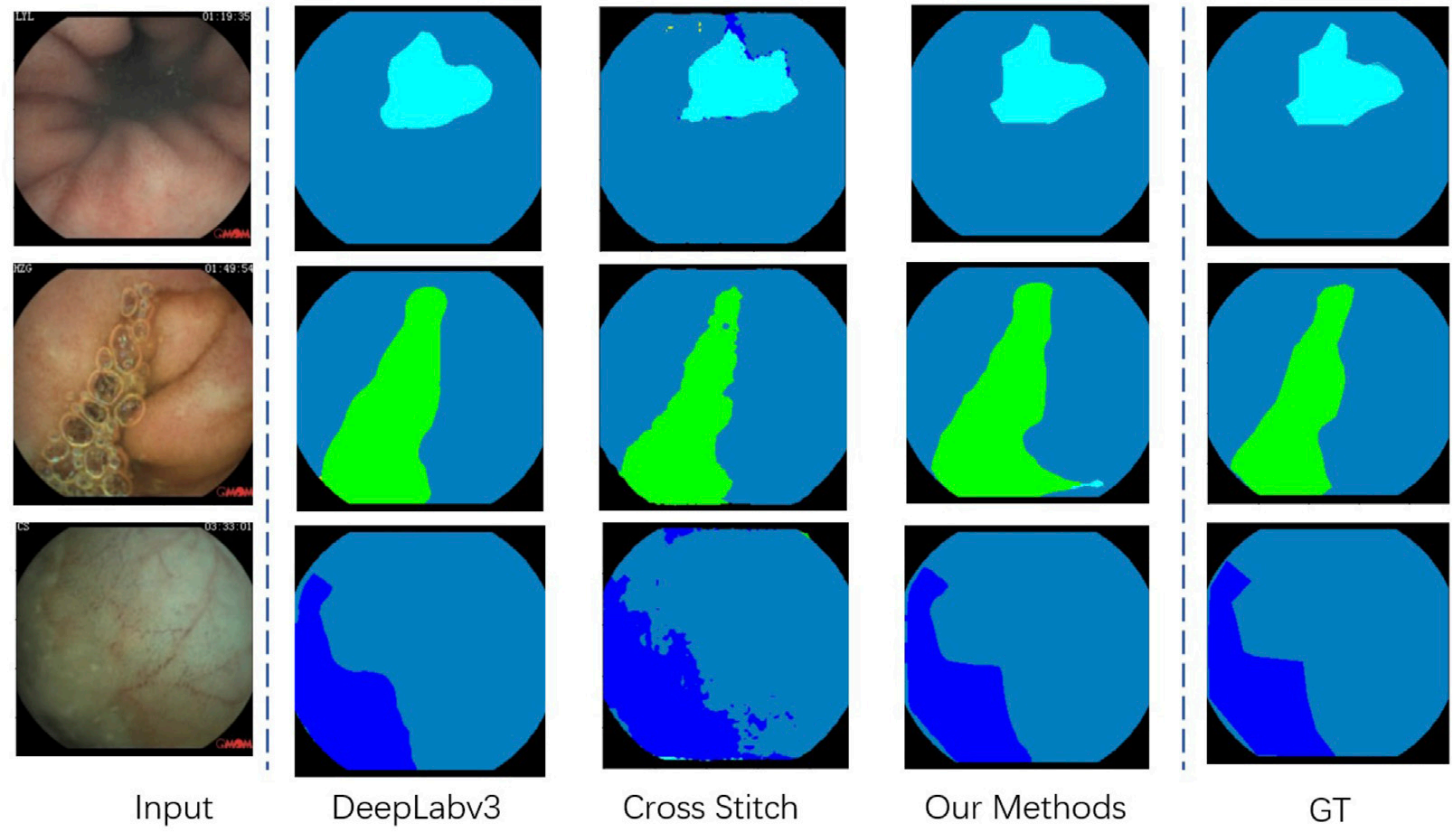
Visualization of the segmentation result in our proposed Capsule-Endoscopy Crohn’s Disease dataset.

• **Single Task, Segmentation Task**. We evaluate four different models on our segmentation problem. Under the same backbone, Table 4 shows that the state-of-the-art segmentation model can achieve competitive results on the CECD dataset. But as shown in Figure 5, the prediction of DeepLabv3 which performs best among them still has huge room for improvement.

**TABLE 4:** Benchmark results in our dataset for the segmentation task.

Segmentation method	Backbone	Iteration	mACC	mIoU
FCN	ResNet-50	30 k	59.5	49.29
PSPNet	ResNet-50	30 k	65.37	54.11
GCNet	ResNet-50	30 k	62.96	53.29
DeepLabv3	ResNet-50	30 k	67.17	54.98

#### 4.3.2 Multi-Task Results

We employ the method described in Section 3.1 and compare it with two widely used multi-task learning methods, and the results are shown in Table 5. Besides, we discuss some structure details when constructing the cross fusion module. We can see from Table 6 that the GAP pooling method in the cross fusion module performs better than GMP. The reason is that the global max pooling may introduce outliers while emphasizing the maximum in cross features.

**TABLE 5:** Detailed analysis of our proposed MTCSN in comparison with others.

Our multi-task method	Backbone	Iteration	Accuracy	Precision	Recall	mACC	mIoU
Hard parameter sharing	ResNet-50	30 k	88.41	80.96	78.93	84.92	77.55
Hard parameter sharing	ResNet-101	30 k	83.3	77.43	77.31	83.08	77.46
Hard parameter sharing	DenseNet-121	30 k	87.5	77.66	78.12	82.08	73.79
Cross stitch	ResNet-50	30 k	80.21	68.92	67.71	81.22	73.33
Cross stitch	ResNet-101	30 k	78.13	73.32	72.51	77.94	69.98
Cross stitch	DenseNet-121	30 k	83.3	72.8	73.09	81.31	74.5
MTCSN	ResNet-101	30 k	**84.75**	**77.78**	**77.91**	**83.27**	75.43
MTCSN	DenseNet-121	30 k	**88.3**	**78.7**	**79.64**	**84.49**	73.75
MTCSN	ResNet-50	30 k	**89.23**	**81.54**	**80.14**	**85.50**	**77.62**

Bold values represents our experiment results suppress all the previous methods.

**TABLE 6:** Ablation studies of the cross fusion module. The global max pooling (GMP) and global average pooling (GAP) denote the different implementation of the cross fusion module on the class fusion branch.

Segmentation method	Accuracy	Precision	Recall	mACC	mIoU
Global max pooling	85.1	73.52	76.1	84.04	72.9
Global average pooling	88.3	78.7	79.64	84.49	73.75

Table 6 shows that our proposed multi-task classification and segmentation network, described in Section 3, achieved the highest performance in both tasks. Because of the imbalance between the two tasks, if we simply apply a multi-task framework, the promotion of segmentation capacity is at the cost of classification performance. Our proposed cross fusion module elegantly fixes the imbalance between them. The qualitative segmentation can also be seen from Figure 5, and the proposed method achieved the best performance.

To the best of our knowledge, no one has previously attempted to utilize segmentation at the pixel level to assist the image-level clearness degree and provide explicable visual results for specialists in clinical practice. In practice, our proposed method will have inference on every frame of the entire output video of capsule endoscopy. The high clearness frames or frames mostly occupied by tissue or lesions will be marked by our framework. In fact, the marked frames only account for 10% of all frames which significantly reduces the heavy work of gastroenterologists. Our pixel-level semantic segmentation results also provide an explicable reference for gastroenterologists to determine the confidence of the output.

## 5 Conclusion

In this work, we propose a multi-task learning framework named the multi-task classification and segmentation network (MTCSN). This framework combines tissue semantic segmentation and clearness degree classification for capsule endoscopy diagnosis. Our MTCSN achieves high performances on both clearness classification tasks and explicable tissue segmentation offering gastroenterologists visualization to understand the whole image. With explicable tissue segmentation, our framework significantly reduces the workload of gastroenterologists and provides steps forward for deep learning–based methods assisting gastroenterologists in clinical practice.

## Data Availability

The raw data supporting the conclusions of this article will be made available by the authors, without undue reservation.
